# Unveiling the impact of tertiary lymphoid structures on immunotherapeutic responses of clear cell renal cell carcinoma

**DOI:** 10.1002/mco2.461

**Published:** 2024-01-12

**Authors:** Wenhao Xu, Jiahe Lu, Xi Tian, Shiqi Ye, Shiyin Wei, Jun Wang, Aihetaimujiang Anwaier, Yuanyuan Qu, Wangrui Liu, Kun Chang, Hailiang Zhang, Dingwei Ye

**Affiliations:** ^1^ Department of Urology Fudan University Shanghai Cancer Center Shanghai China; ^2^ Department of Oncology Shanghai Medical College Fudan University Shanghai China; ^3^ Shanghai Genitourinary Cancer Institute Shanghai China; ^4^ School of Cellular and Molecular Medicine University of Bristol Bristol UK; ^5^ Affiliated Hospital of Youjiang Medical University for Nationalities Baise China; ^6^ State Key Laboratory of Oncology in South China Collaborative Innovation Center for Cancer Medicine Department of Urology Sun Yat‐sen University Cancer Center Guangzhou China; ^7^ Renji Hospital Shanghai Jiao Tong University School of Medicine Shanghai China

**Keywords:** clear cell renal cell carcinoma, immune checkpoint inhibitors, tertiary lymphoid structures, tumor heterogeneity, tumor microenvironment

## Abstract

Tertiary lymphoid structures (TLS) are organized aggregates of immune cells that form under pathological conditions. However, the predictive value of TLS in clear cell renal cell carcinoma (ccRCC) for immunotherapies remains unclear. We comprehensively assessed the implications for prognosis and immunological responses of the TLS spatial and maturation heterogeneity in 655 ccRCC patients. A higher proportion of early‐TLS was found in peritumoral TLS, while intratumoral TLS mainly comprised secondary follicle‐like TLS (SFL‐TLS), indicating markedly better survival. Notably, presence of TLS, especially intratumoral TLS and SFL‐TLS, significantly correlated with better survival and objective reflection rate for ccRCC patients receiving anti‐Programmed Cell Death Protein‐1 (PD‐1)/Programmed Cell Death‐Ligand‐1 (PD‐L1) immunotherapies. In peritumoral TLS cluster, primary follicle‐like TLS, the proportion of tumor‐associated macrophages, and Treg infiltration in the peritumoral regions increased prominently, suggesting an immunosuppressive tumor microenvironment. Interestingly, spatial transcriptome annotation and multispectral fluorescence showed that an abundance of mature plasma cells within mature TLS has the capacity to produce IgA and IgG, which demonstrate significantly higher objective response rates and a superior prognosis for ccRCC patients subjected to immunotherapy. In conclusion, this study revealed the implications of TLS spatial and maturation heterogeneity on the immunological status and clinical responses, allowing the improvement of precise immunotherapies of ccRCC.

## Graphical abstract

H&E, IHC, mIHC, and spatial transcriptomics were implemented to analyze spatial and maturation heterogeneity of tertiary lymphoid structures (TLS) in ccRCC. Mature TLS within tumors participate in antitumor immunity and might serve as the locale for the generation of IgA and IgG plasma cell, predicting favorable prognosis and effectiveness of immunotherapy, whereas peritumoral immature TLS show opposite effects for ccRCC patients subjected to immunotherapy.

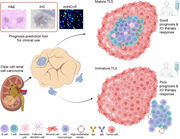

## 1 INTRODUCTION

Clear cell renal cell carcinoma (ccRCC) is the most common and malignant histological type of kidney cancer in adults, accounting for over 75% of all kidney cancers.^1^, ^2^, ^3^, ^4^ ccRCC is typically sensitive to immune checkpoint inhibitors (ICIs) therapy, such as Programmed Cell Death Protein‐1 (PD‐1) and Cytotoxic T Lymphcyte‐Associated Antigen‐4 (CTLA‐4) inhibitors, particularly for restraining its progression, relapse, and metastasis.^5^, ^6^, ^7^ However, ccRCC is a malignancy with high heterogeneity, and tumor cells with distinct genetic phenotypes and mutation profiles lead to diverse therapeutic sensitivities and prognosis^8^, ^9^, ^10^ The screening and identification of efficient biomarkers for prognostic prediction, treatment selection, and efficacy evaluation have become one of the biggest challenges to the current diagnosis and treatment given the environment of ccRCC.^11^, ^12^ Consequently, determining the biomarker utility of mapping cellular organization of the ccRCC microenvironment merits investigation.

Tertiary lymphoid structure (TLS) are a pathological phenomenon that is distinct from primary and secondary lymphoid organs (SLOs). They are the aggregates of immune cells formed in tissues in response to chronic inflammation or persistent immune stimulation, such as tumor. TLS can be further classified into three maturation stages, early‐TLS (E‐TLS), primary follicle‐like TLS (PFL‐TLS), and secondary follicle‐like TLS (SFL‐TLS), based on their cellular composition; or two groups, peritumoral TLS and intratumoral TLS (intra‐TLS), based on its relative localization to the tumor.^13^ TLS induce antitumor responses and the heterogeneity of TLS is associated with different levels of immune response to the tumor and contradictory prognosis of patients.^14^ For example, immature TLS, are unstructured T or B cell aggregates that may not be sufficient to induce antitumor reactions and are found associated with immune suppressive tumor microenvironment (TME) in some tumors.^15^, ^16^, ^17^ While the more organized, mature TLS, featured by CD21^+^ CD23^+/−^ follicular dendritic cells (FDCs), B cell lymphoma 6 (BCL6^+^) germinal center (GC) B cells and plasma cells, are able to generate “in situ” immunity against tumors, likely through antibody‐induced apoptosis.^14^ Researches showed that B cell undergo isotype switching and hypermutation, so that the high‐affinity antibody‐producing cells were selected and even infiltrate into tumor.^18^ Therefore, investigating the prognostic value and immune function of TLS in ccRCC is of our great interest, though the mechanism of the initiation and maturation of TLS in ccRCC is not fully elucidated.

The TME is an evolving concept that defines the behavior of cancers not only by the genetics of tumor cells but also by the surrounding environment that the tumor cells need to survive, proliferate, and metastasize.^19^ The immunosuppressive TME of the ccRCC is infiltrated with a substantial amount of immune cells, primarily CD8^+^ T cells, with the addition of tumor‐associated macrophages (TAMs), natural killer (NK) cells, and B cells.^20^, ^21^ Work from Haydn T. Kissick's lab identified an antigen‐presenting cell (APC)‐dense niche within kidney tumors that harbors stem‐like CD8^+^ T cells, allowing them to differentiate into the terminally differentiated PD‐1^+^ TIM3^+^ T cells. This process of T cell development leads to a higher percentage of T cell infiltration as well as a better prognosis and response rate to ICIs therapy.^22^ The absence or loss of such APC‐niche is associated with kidney tumor progression. Moreover, a TME rich in CD8 T cells expressing a high level of PD‐1 but missing fully mature dendritic cells (DCs) is linked to a higher risk of ccRCC advancement. In contrast, the presence of mature DCs at the TLS in the periphery of the tumor with activated CD8^+^ T cells indicates a better prognosis.^23^ In the setting of tumors, TLS facilitate the influx of immune cells into the TME sites and have therefore attracted interest as potential mediators of improving immunotherapies and predicting prognosis for patients. Despite of their presumed importance, the drivers of tumor‐specific TLS maturation and the contribution of TLS heterogeneity to clinical outcomes and TME characterizations in ccRCC remain incompletely understood. Overall, investigating the immune cell organization in the ccRCC TME will bring valuable insights to the decision‐making of immunotherapies.

Immunotherapy has emerged as a promising approach in cancer treatment, harnessing the body's immune system to target tumor cells. TLS are thought to play a role in the tumor‐specific immune response to solid tumors^24^ and are related to better prognosis and response to immunotherapy in many cancer types, including ovarian, lung, colorectal, and bladder cancers.^13^ However, the presence of TLS and their impact on ccRCC prognosis and response to immunotherapy are controversial.^18^, ^25^ This study hypothesized that this heterogeneity in prognosis may be due to different maturation statuses and localizations of TLS. Identification of SFL‐TLS located inside the tumor bears clinical significance, while peritumor TLS at immature stages indicate a poor prognosis.

## 2 RESULTS

### 2.1 Clinicopathological characteristics for patients with ccRCC from the integrated Fudan University Shanghai Cancer Center cohorts

The baseline clinicopathological characteristics of 429 ccRCC patients from the retrospective integrated Fudan University Shanghai Cancer Center (FUSCC) cohorts are summarized in Table S1. In the integrated FUSCC cohorts, for patients with ccRCC, until the last day of follow‐up (October 30, 2023), the median progression‐free survival (PFS) and overall survival (OS) were 43.5 and 65.3 months, respectively. 62.5% of patients were male. The median age was 55 years, ranging from 20 to 86 years. Among the traditional clinical and pathological parameters, 24.0% patients were in T3–T4 stage, 20.0% patients were in N1 stage, 15.6% patients were in M1 stage, 28.4% patients were in advanced American Joint Committee on Cancer (AJCC) stage, and 43.8% patients were in aggressive International Society of Urological Pathology (ISUP) grade at the first diagnosis.

### 2.2 Morphological structure of tumor‐associated TLS in ccRCC

In this study focusing on ccRCC, all included tissue samples underwent immunohistochemical staining following Hematoxylin and eosin (H&E) staining, aimed at identifying potential TLS and evaluating maturity and spatial heterogeneity. The presence, localization (intratumoral or peritumoral), and maturation (E‐TLS, PFL‐TLS, or SFL‐TLS) of TLS were initially assessed based on the clinicopathological characteristics analyzed by H&E staining and confirmed by immunohistochemistry (IHC) staining for primary ccRCC tissues for diagnoses cases of 429 patients from integrated FUSCC cohorts. Within the retrospective cohort, 166 specimens exhibited potential microscopic TLS after H&E staining, ultimately culminating in 145 specimens confirmed to bear definite TLS presence via IHC identification (33.8%) (Table S1). Remarkably, only a minority (6.6%) of initially suspected TLS specimens lacked classical TLS structures, displaying mere immune and stromal cell aggregation. This suggests the practical feasibility and clinical applicability of utilizing H&E staining for preliminary TLS identification in large‐scale sample sets, thereby establishing a groundwork for subsequent clinicopathological investigations related to tumor‐associated TLS (TA‐TLS). This approach underlines the importance of H&E staining in determining the accuracy and sensitivity of TA‐TLS identification.

**FIGURE 1:**
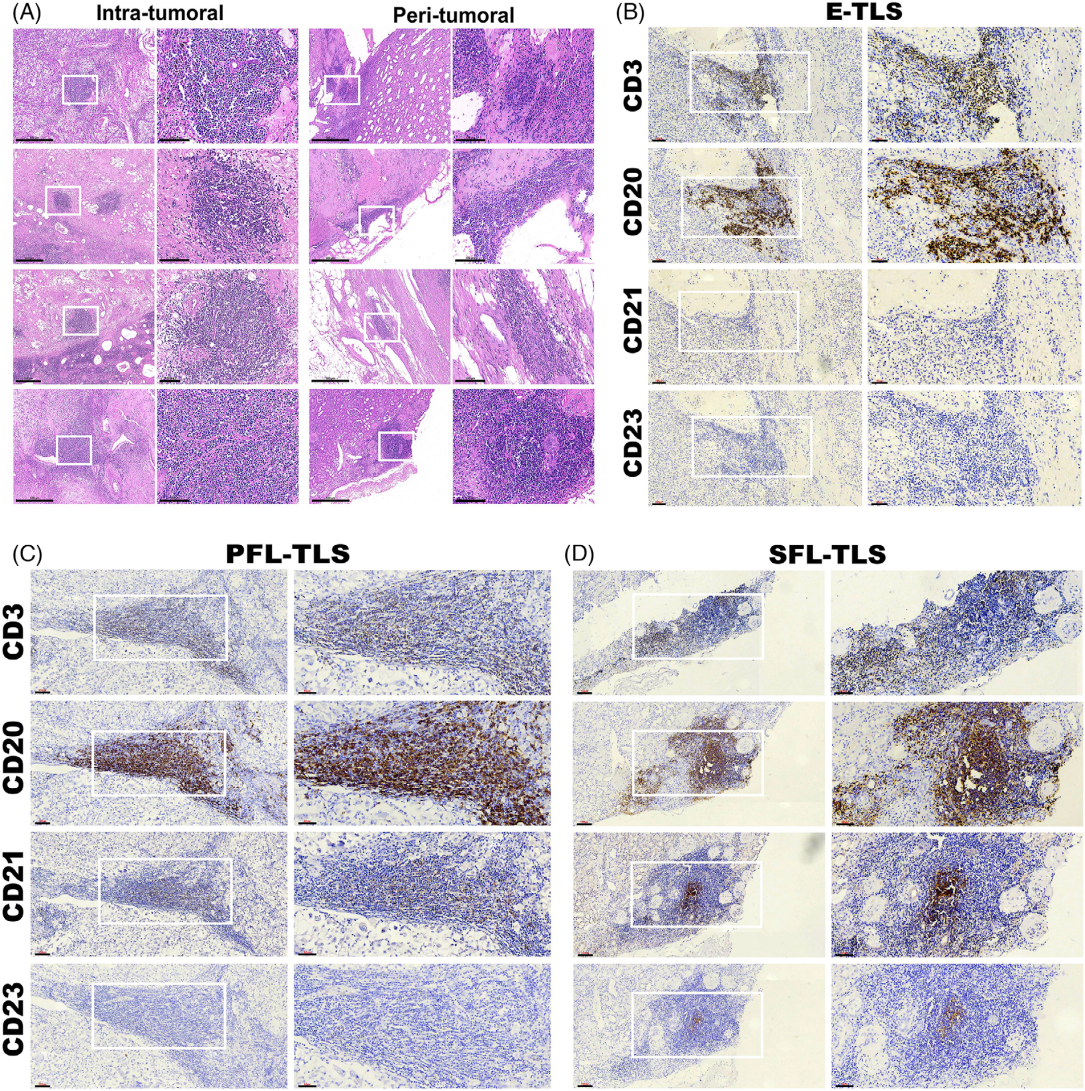
Representative structure of tertiary lymphoid structures (TLSs) in clear cell renal cell carcinoma (ccRCC). (A) Representative H&E staining images of intratumoral and peritumoral TLSs in ccRCC. Scale bars: (large field) 500 μm, (zoomed in) 100 μm. (B–D) Representative immunohistochemistry (IHC) staining images of E‐TLS (B), PFL‐TLS (C), and SFL‐TLS (D) in ccRCC combining CD3 (for CD3^+^ T cells), CD20 (for CD20^+^ follicular B cells), CD21 (for CD21^+^ follicular dendritic cells), and CD23 (CD23^+^ germinal center cells). E‐TLS, early‐TLS; PFL‐TLS, primary follicle‐like TLS; SFL‐TLS, secondary follicle‐like TLS. Scale bars: (large field) 100 μm, (zoomed in) 60 μm.

Specifically, the majority of TLS exhibited an irregular nest‐like structure, often presenting as circular dense regions (Figures 1A and S1). Notably, the size and shape of TLS appeared consistent across different spatial contexts, indicating a degree of uniformity irrespective of spatial heterogeneity. However, our observations suggest a correlation between the maturation stage of TLS and the area covered, hinting at a potential link between larger areas and a longer formation time attributed to the development of a more extensive array of cellular components within mature TLS.

Previous reports have postulated that the association of the two subtypes of TLS localization with disease progression could differ.^26^, ^27^ In most cancers, no clear relationship between the peritumoral or intratumoral location of TLS and clinical outcomes has been demonstrated. To define the maturation of TLS in ccRCC, we implemented an IHC assay to evaluate aggregates of lymphocytes that have histological features with analogous structures to lymphoid tissues with CD3^+^ T cells, CD20^+^ follicular B cells, CD21^+^ FDCs infiltration, and CD23^+^ GC cells in serial sections (Figures 1B–D). Analogous to the stages of SLOs follicles, we defined lymphocytic clusters without FDC as E‐TLS (the first phase of TLS maturation), FDC‐existing TLS without GC as PFL‐TLS (the transitional phase of TLS maturation), and GC‐existing as SFL‐TLS (the final phase of TLS maturation, mature TLS identified for ccRCC).^28^

Further characterization of TLS structural attributes revealed a consistent organization that included distinctive B cell and T cell regions among all the maturation stage of TLS (Figures 1B–D). Notably, CD20^+^ B cells occupied the inner regions, encircled by CD3^+^ T cells, forming structures reminiscent of lymphoid follicles akin to SLOs. Additionally, the presence of DCs and GC within these structures, alongside the representation of maturity through various markers CD21 and CD23, provided valuable insights into delineating the maturity stages of TLS associated with ccRCC tumors. These observations serve to shed light on the complex architecture and maturity states of TLS within the ccRCC microenvironment, emphasizing their potential impact on the tumor's immune responses and microenvironment features.

### 2.3 Characteristics of TLS localization and maturation heterogeneity in ccRCC

**FIGURE 2:**
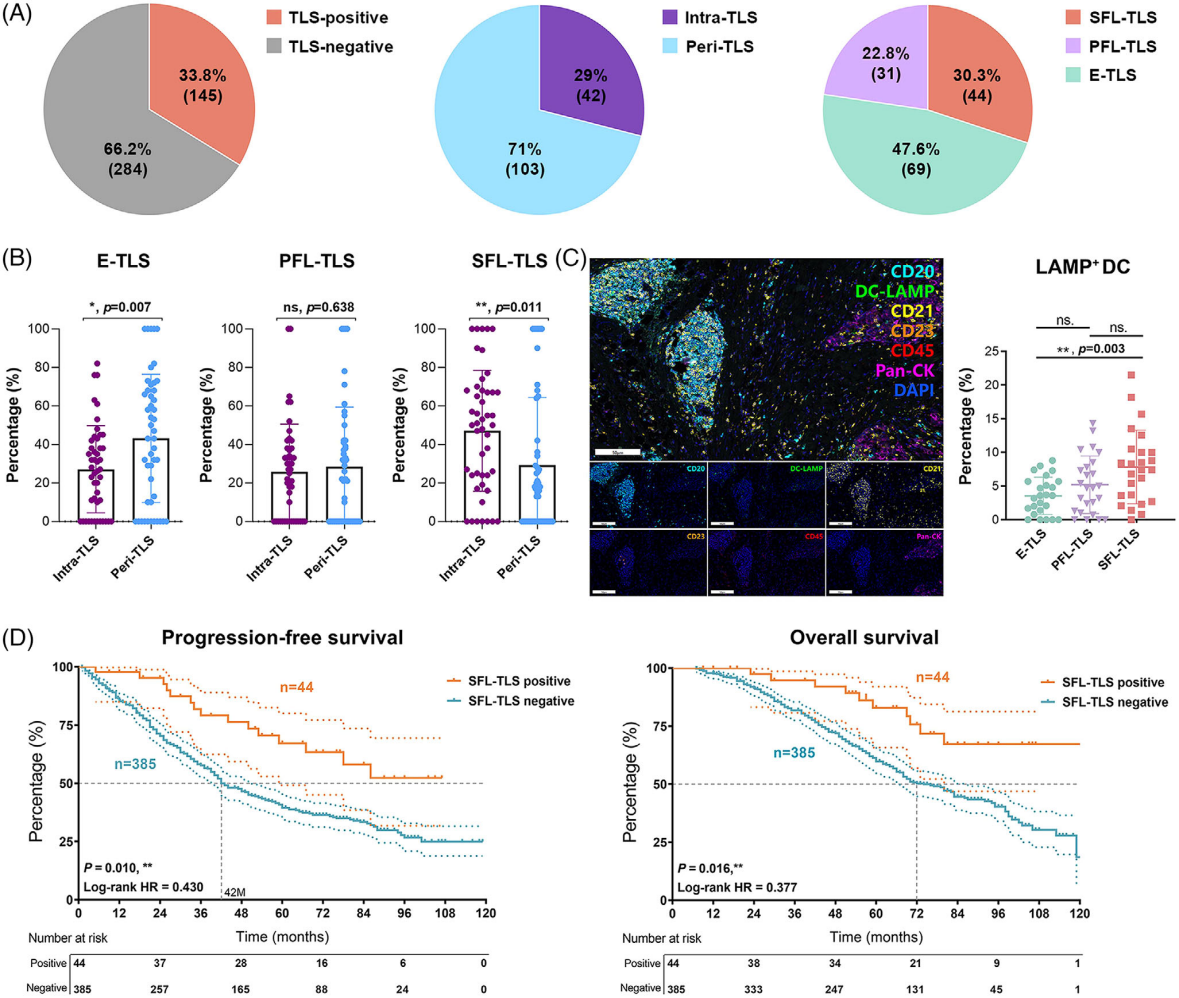
Characteristics of TLS localization and maturation heterogeneity in ccRCC. (A) The statistic characteristics of TLS location and maturation in the integrated FUSCC cohorts comprised ccRCC 429 patients. (B) Percentage of E‐TLS, PFL‐TLS, and SFL‐TLS in ccRCC intratumoral and peritumoral TLSs. (C) Representative seven‐color multispectral immunofluorescence (mIF) staining images of ccRCC tissues combining CD20, DC‐LAMP, CD21, CD23, CD45, Pan‐CK, and DAPI, and the percentage of LAMP^+^DC in E‐TLS, PFL‐TLS, and SFL‐TLS of ccRCC. (D) Kaplan–Meier curve comparing overall survival (OS) and progression‐free survival (PFS) between SFL‐TLS positive and negative ccRCC patients.

In the cohort of specimens subjected to continuous section IHC staining, analysis revealed distinct classifications based on the localization and maturation heterogeneity of TLS within the context of ccRCC. Among the specimens studied, 103 were identified to solely exhibit tumor‐distal TLS, thereby categorized as peritumoral TLS (peri‐TLS, constituting 71.0% of the samples). In contrast, 42 samples demonstrated at least one tumor‐proximal TLS, thereby classified as intra‐TLS (accounting for 29.0% of the cases) (Figure 2A).

In terms of maturation, the observed TLS populations exhibited diverse characteristics: 44 cases (30.3%) displayed at least one SFL‐TLS, characterized by the presence of both FDC and GC reactions, thus forming the mature TLS group. Furthermore, 31 cases (22.8%) showcased at least one PFL‐TLS, while 69 cases (47.6%) exclusively manifested early follicle‐like TLS (E‐TLS and PFL‐TLS), devoid of both FDC and GC reactions (Figure 2A). The latter two groups, categorized as immature TLS groups, exhibited distinct stages of TLS maturation within the ccRCC microenvironment. These defined classifications based on the localization and maturity of TLS provide an intricate and segmented view of TLS diversity within the ccRCC setting, establishing a foundation for understanding their potential role in influencing immune microenvironment features.

Then, we conducted an analysis to investigate the relationship between TLS localization heterogeneity and maturation stages. We aimed to compare the proportions of TLS at different maturation stages within the intratumoral and peritumoral TLS groups. Our findings revealed notable differences: the proportion of E‐TLS in the peri‐TLS cluster significantly surpassed that in the intra‐TLS group (*p* = 0.017). Conversely, the proportion of mature SFL‐TLS in the intra‐TLS cluster markedly exceeded that in the peri‐TLS cluster (*p* = 0.011; Figure 2B). These observations indicate that while the proportion of intra‐TLS within the TME might be relatively low, the morphology of intra‐TLS in these locations tends to exhibit higher maturity. This suggests a potentially critical role in stable antigen presentation, fostering mature cell interactions, and facilitating immune responses against the tumor. The distinct differences in TLS maturity between intratumoral and peritumoral clusters highlight their potential functional variances and contributions to the immune landscape within the context of ccRCC.

For the effective recognition of pathogens and the initiation of a normal immune response, the pivotal role of APCs in presenting antigens is fundamental. Particularly, the antigen‐presenting ability of DCs is intricately linked to their state of maturity. Mature antigen‐presenting DCs, denoted by the expression of DC‐LAMP, demonstrate robust antigen‐presenting capabilities. These mature DCs are prominently detected during the developing maturation phases of TLS, indicating a strong aptitude for antigen presentation within these specialized structures. Given that the infiltration of LAMP+DCs is associated with enhanced clinical survival, we employed seven‐color multispectral immunofluorescence (mIF) to investigate whether these specific cell types could serve as predictive markers for distinct stages of TLS maturation (Figure 2C). Our analysis revealed a substantial increase in the LAMP^+^DC ratio concurrent with the advancement of TLS maturity stages. Notably, this ratio reached its peak within the SFL‐TLS cluster, significantly surpassing that observed in the immature E‐TLS (*p* = 0.003). These findings underscore the association between the presence of mature DCs within TLS and the TLS maturation, emphasizing their potential impact on antigen presentation and modulation of immune responses within the milieu of ccRCC.

To substantiate the antitumor immune effect attributed to the SFL‐TLS, characterized by their mature and stable functionality, we isolated the SFL‐TLS cluster group from the integrated FUSCC cohort. We then conducted a comparative survival analysis between this cluster and the entirety of samples devoid of SFL‐TLS within the retrospective cohort (Figure 2D). Our survival analysis outcomes revealed a significant disparity in both PFS and OS between the SFL‐TLS positive group and the negative group. Specifically, the group with SFL‐TLS demonstrated markedly superior PFS (*p* = 0.010, log‐rank HR = 0.430) and OS (*p* = 0.016, log‐rank HR = 0.377) outcomes compared with those without SFL‐TLS.

### 2.4 Spatial transcriptomics analysis of TLS heterogeneity in ccRCC

**FIGURE 3:**
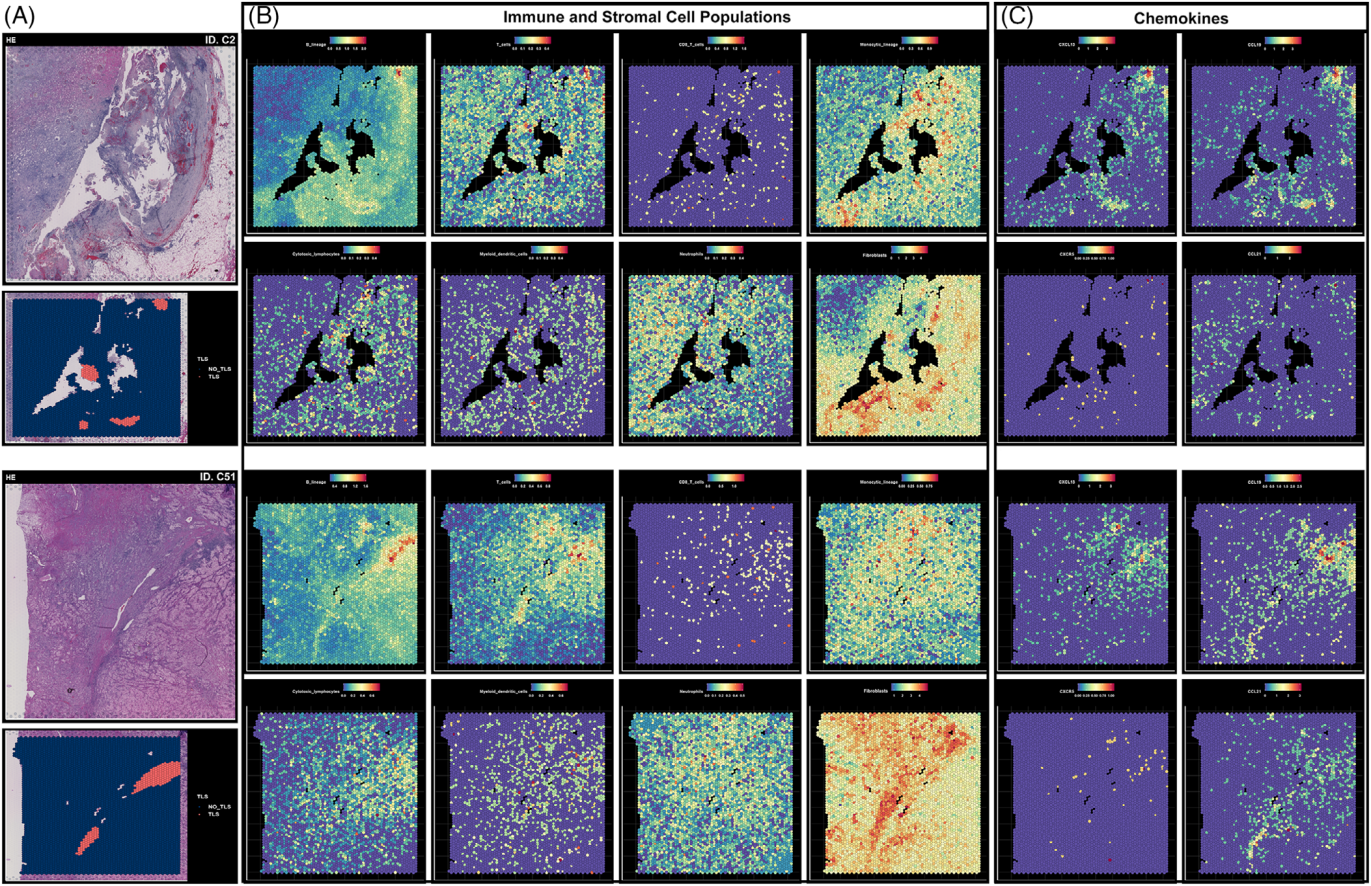
The TLS heterogeneity in ccRCC revealed by spatial transcriptomics analysis. (A) The H&E staining images and TLS annotations (reported by Maxime Meylan) in two ccRCC samples (ID. C2 and C51). (B) The location and abundance of immune and stroma cell populations. (C) The location and abundance of prominently TLS‐related chemokines, including CXCL13, CXCL5, CCL19, and CCL21.

Based on the TLS annotation, we explored the location of TLS in the TME. In Figure 3A, we displayed images of sections stained with H&E, with TLS annotations provided by Maxime Meylan's research highlighted in red. We quantified the lymphocytes abundance and explored the expression level of various chemokines. As depicted in Figure 3B, the expression of gene signatures specific to immune and stromal cell populations, especially B lineage, T cells, and fibroblasts, was predominantly observed in hot spots within the TLS regions. In these two specimens, the CD8^+^ T cell count was relatively low, while the monocytic lineage and cytotoxic lymphocytes showed a slight increase within the TLS structure. Myeloid DCs and neutrophils appeared to be evenly distributed and did not show significant enrichment near the TLS structure. Near the TLS structure, there was a significant increase in the expression of genes closely associated with fibroblasts, suggesting a potential role for fibroblasts in the formation and function of TLS. Additionally, the prominent distribution of TLS‐related chemokines, including CXCL13, CXCL5, CCL19, and CCL21, is depicted in Figure 3C. Significantly strong enrichment of CXCL13, CCL19, and CCL21 was observed within the TLS localization regions in these detected ccRCC specimens, while the potential relationship between expression of CXCR5 and TLS location was not functionally observed.

### 2.5 Effect of heterogeneity in TLS spatial localization and maturation on TME status of ccRCC

Previous studies have substantiated the existence of three stages of TLS maturation within the TME, and their close association with disease recurrence and the long‐term prognosis of tumor patients.^28^, ^29^ A more comprehensive characterization of TLS heterogeneity is anticipated to aid in defining tumor immunophenotypes based on TLS status, encompassing cell composition, spatial location, maturation, and the functional aspects of antitumor immune responses.

**FIGURE 4:**
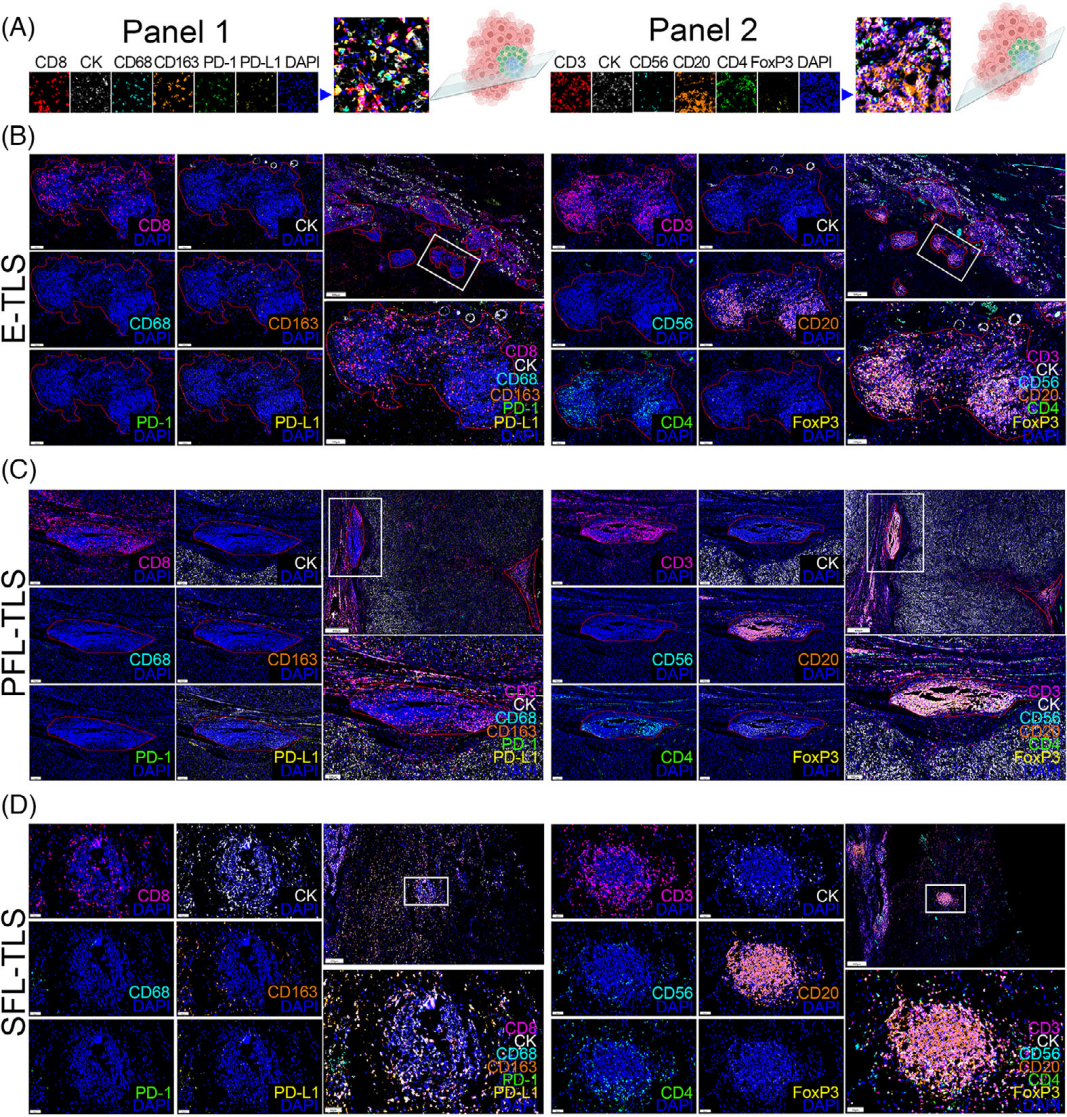
Effect of heterogeneity in TLS spatial localization and maturation on ccRCC TME assessed by multispectral fluorescence immunohistochemistry (mIHC). (A) The cellular composition of TLS was detected by mIHC in 63 cases of ccRCC tissues using two panels of 12 antibody markers. (B–D) Representative mIHC staining images of ccRCC tissues depicting the differences in tumor‐infiltrated lymphocytes among E‐TLS, PFL‐TLS, and SFL‐TLS. Scale bars: (B) (large field) 500 μm, (zoomed in) 100 μm; (C) (large field) 400 μm, (zoomed in) 100 μm; (D) (large field) left: 250 μm, right: 400 μm, (zoomed in) 50 μm.

To obtain a comprehensive understanding of TLS heterogeneity within heterogeneous ccRCC samples, we performed mIHC staining on 63 samples. The analyses focused on delineating the cellular composition profiles within TLS at different stages of maturation in ccRCC tissues. The mIHC technique utilized two seven‐color combinations (Panel 1: CD8, CK, CD68, CD163, PD‐1, PD‐L1, DAPI; Panel 2: CD3, CK, CD56, CD20, CD4, FoxP3, and DAPI), as illustrated in Figure 4A, generating a total of 12 primary antibody staining images. The overall mIHC staining image was demonstrated at a low power microscope (Figure S2). The analyses of cellular composition at different TLS maturation stages aimed to unveil the intricacies of TLS heterogeneity and its impact on the local TME of ccRCC. For instance, Figures 4B–D delineate the cellular compositions of E‐TLS, PFL‐TLS, and SFL‐TLS, respectively.

**FIGURE 5:**
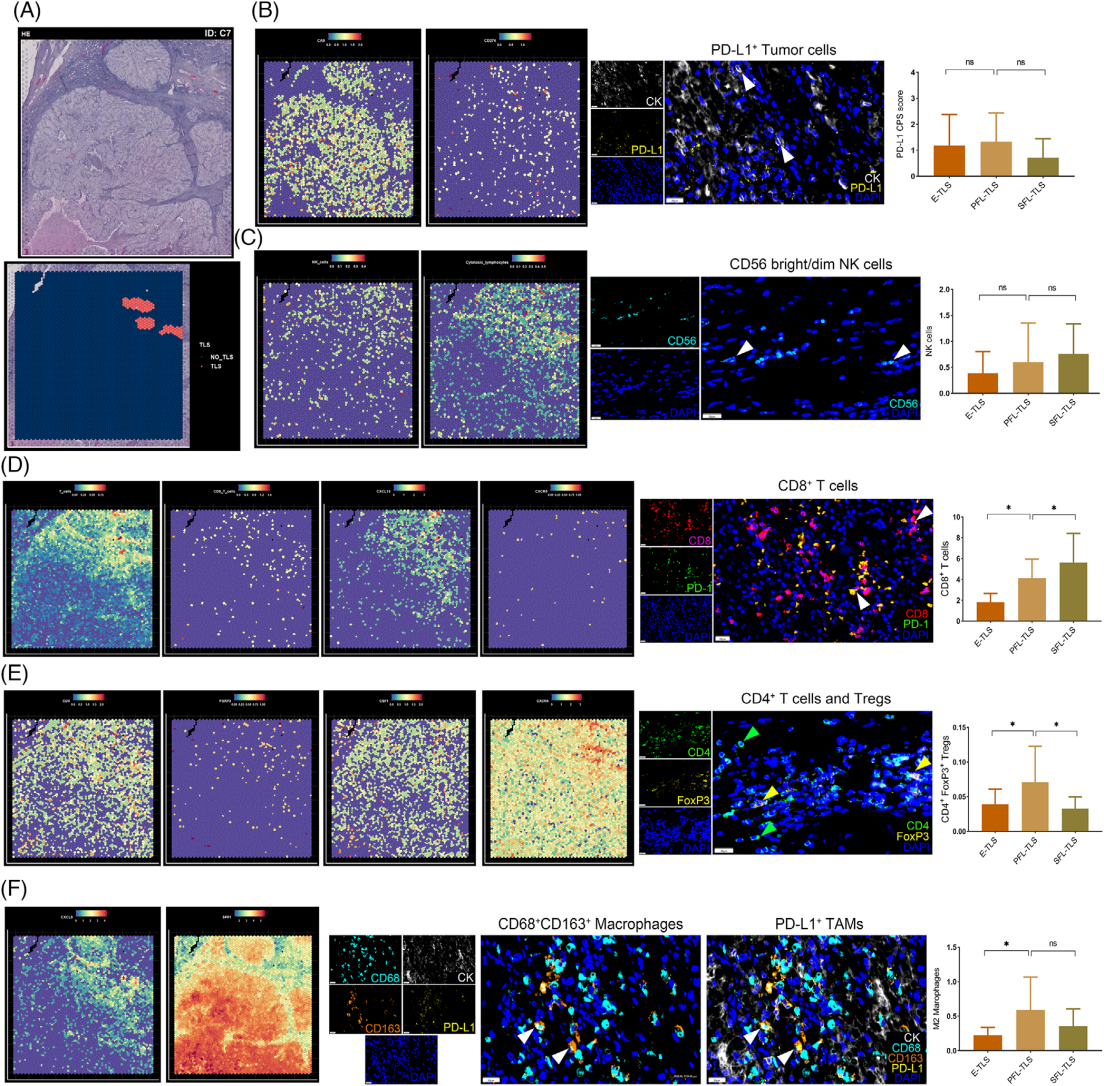
Spatial transcriptomics analysis revealed the effect of TLS heterogeneity on ccRCC TME. (A) The H&E staining images and TLS annotations (reported by Maxime Meylan) of a ccRCC sample (ID. C7) with spatially defined transcriptome data. (B) The correlation of location between PD‐L1^+^ tumor cells and TLS. The spatial location of CA9 and CD274 (left), the representative mIF staining images of CK, PD‐L1, and DAPI (middle), and PD‐L1 CPS scores in E‐TLS, PFL‐TLS, and SFL‐TLS (right). (C) The correlation of location between NK cells and TLS. The spatial location of NK cells and cytotoxic lymphocytes (left), the representative mIF staining images of CD56 and DAPI (middle), and the abundance of NK cells in E‐TLS, PFL‐TLS, and SFL‐TLS (right). (D) The correlation of location between T cells and TLS. The spatial location of T cells, CD8^+^ T cells, CSCL13, and CSCR5 (left), the representative mIF staining images of CD8, PD‐1, and DAPI (middle), and the abundance of CD8^+^ T cells in E‐TLS, PFL‐TLS, and SFL‐TLS (right). (E) Evaluation immunosuppressive TME statuses among TLS at varying maturation stages. The spatial location of CD4, FoxP3, CSF1, and CXCR4 (left), the representative mIF staining images of CD4, FoxP3, and DAPI (middle), and the abundance of CD4^+^FoxP3^+^ T cells in E‐TLS, PFL‐TLS, and SFL‐TLS (right). (F) The correlation of location between macrophages and TLS. The spatial location of CXCL9 and SPP1 (left), the representative mIF staining images of CD68, CK, CD163, PD‐L1, and DAPI (middle), and the abundance of M2‐macrophages in E‐TLS, PFL‐TLS, and SFL‐TLS (right).

Moreover, we utilized spatial transcriptome data in combination with mIHC to quantitatively evaluate the extent of multiple immune cell infiltrations. For instance, Figure 5A showcases the spatially defined transcriptome data within the TME based on Meylan's cohort, outlining the location of TLS in the H&E profile of a sample The spatially‐resolved transcriptomic data revealed intricate associations and disassociations. For instance, the analysis of PD‐L1 expression, defined by the expressions of carbonic anhydrase 9 (CA9) and CD274, indicated no close relationship with the spatial location of TLS (Figure 5B). Additionally, the spatial location and maturity of CD56^+^ NK cells within TLS did not exhibit a significant correlation, while cytotoxic lymphocytes were mainly abundant in TLS spatial regions (Figure 5C). Furthermore, our results indicated that T cells were highly infiltrated in the spatial region surrounding TLS, displaying significant associations with CXCL13 enrichment, yet not with CXCR5 enrichment (Figure 5D). Interestingly, as evaluated through mIF, the proportion of CD8^+^ T cells within TLS increased in tandem with the maturity stage of TLS, with significantly higher proportions observed in SFL‐TLS compared with immature TLS groups. This finding suggests a potential association between TLS maturity stages and antitumor immune response effectiveness.

Intriguingly, the analysis extended to the evaluation of different immunosuppressive TME statuses among TLS at varying maturation stages. Spatial transcriptomic information suggested that CD4^+^CXCR4^+^ T cells were highly enriched in the TLS region (Figure 5E). Additionally, mIHC analyses revealed significantly higher proportions of CD4^+^FoxP3^+^ regulatory T cells (Tregs) in immature TLS compared with mature stages, particularly peaking in the PFL‐TLS cluster group (Figure 5E). Moreover, the investigation into the relationship between the presence and polarization status of TAMs and TLS heterogeneity disclosed compelling insights. The expression of CXCL9 and SPP1 was employed to define TAM polarization, characterizing their roles in tumor promotion or suppression. Our results indicated the dominance of M1 macrophages in TA‐TLS; however, a significant number of macrophages in an immunosuppressive M2 polarization state surrounded the TLS spatial region (Figure 5F). Moreover, we observed significantly higher proportions of immunosuppressive M2 macrophages in immature TLS compared with mature SFL‐TLS clusters. These findings emphasize the potential of mature TLS to circumvent the “immune blockade” and exert a more stable antitumor immune effect compared with their immature counterparts, which exhibit an immunosuppressive TME state of ccRCC.

### 2.6 TLS heterogeneity predicts clinical outcomes and treatment responses for ccRCC patients receiving immunotherapies

The latest RCC guidelines recommend tyrosine kinase inhibitors (TKIs) and ICIs combination therapy as the conventional first‐line therapy for metastatic ccRCC patients. To delve into the potential correlation between the heterogeneity of TLS and the response to immunotherapy, our study engaged 230 patients diagnosed with advanced ccRCC who underwent postoperative adjuvant therapy involving anti‐PD‐1/PD‐L1 combined with TKIs therapy from the FU‐ICIs cohort. In our analysis, among the various ccRCC tissue samples exhibiting TLS presence, we stratified them based on the types of TLS observed. Specifically, the samples were categorized into distinct groups: the intra‐/mature group encompassed samples displaying at least one intratumoral TLS and at least one SFL‐TLS, while the intra‐/immature group comprised samples with solely intratumoral TLS and lacking SFL‐TLS. Conversely, the peri‐/mature group included samples demonstrating only peritumoral TLS along with at least one SFL‐TLS, while the peri‐/immature group consisted of samples with solely peritumoral TLS and no SFL‐TLS.

**FIGURE 6:**
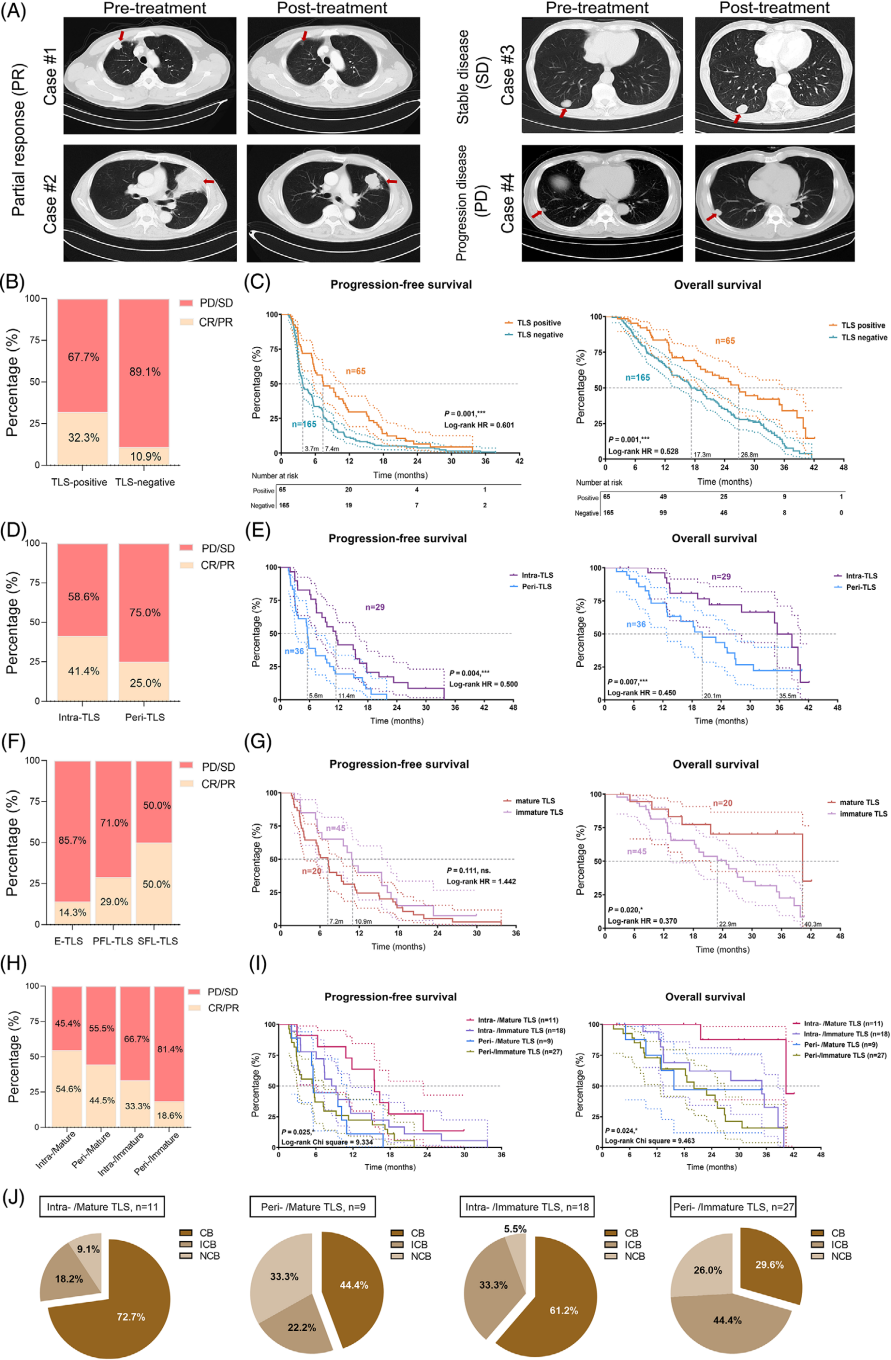
Utilization of TLS heterogeneity to predict clinical outcomes and immunotherapy response in ccRCC patients. (A) The representative chest CT images of metastatic ccRCC patients with different objective response rate (PR, SD, and PD) in FU‐ICIs cohort. PR, partial response; SD, stable disease; PD, progressive disease. (B) The percentage of PD/SD and CR/PR metastatic ccRCC patients in TLS‐positive and TLS‐negative groups. (C) Kaplan–Meier curve comparing OS and PFS between TLS‐positive and TLS‐negative ccRCC patients. (D) The percentage of PD/SD and CR/PR metastatic ccRCC patients in intra‐TLS and peri‐TLS groups. (E) Kaplan–Meier curve comparing OS and PFS between intra‐TLS and peri‐TLS ccRCC patients. (F) The percentage of PD/SD and CR/PR metastatic ccRCC patients in E‐TLS, PFL‐TLS, and SFL‐TLS groups. (G) Kaplan–Meier curve comparing OS and PFS between mature‐TLS and immature‐TLS groups. (H) The percentage of PD/SD and CR/PR metastatic ccRCC patients in peri‐/immature, intra‐/immature, peri‐/mature, and intra‐/mature groups. (I) Kaplan–Meier curve comparing OS and PFS between peri‐/immature, intra‐/immature, peri‐/mature, and intra‐/mature groups. (J) The clinical benefit analysis in ccRCC patient groups following ICIs treatment. CB, clinical benefit; ICB, intermediate clinical benefit; NCB, no clinical benefit.

As shown in Figure 6A, FU‐ICIs cohort showed that metastatic ccRCC patients exhibited a variety of therapeutic effects and objective response rate, including complete response (CR), partial response (PR), progressive disease (PD), and stable disease (SD), which was evaluated by the FUSCC clinician using enhanced computed tomography imaging. For 230 ccRCC patients, 28.3% samples (*n* = 65) was identified with presence of TLSs using IHC staining. The number of patients with TLS‐positive clusters responses to TKIs+ICIs were considerably elevated than patients with TLS‐negative clusters (32.3 vs. 10.9%; Figure 6B). Patients with TLS‐positive in FU‐ICIs cohort had a prominently longer PFS (*p* = 0.001, HR = 0.601) and OS than those samples without TLS presence (*p* = 0.001, HR = 0.528; Figure 6C). To be specific, we found significant differential objective reflection rate between patients with intratumoral and peritumoral TLS (41.4 vs. 25.0% in CR/PR; Figure 6D). Patients with intratumoral TLS also exerted significantly elevated PFS (*p* = 0.004, HR = 0.500) and OS (*p* = 0.007, HR = 0.450) than patients in peri‐TLS cluster (Figure 6E). Among the TLS maturation stages, we found the percent of samples undergoing CR/PR responses increased with heterogeneity of maturation TLS: 14.3% in E‐TLS clusters, 29.0% in PFL‐TLS clusters, and 50.0% in SFL‐TLS clusters (Figure 6F). Besides, although mature TLS (namely SFL‐TLS, with CD23^+^ GC cells) fail to exert significant differences in PFS (*p* = 0.111), while predicted remarkably elevated OS (*p* = 0.020, HR = 0.370) than ccRCC patients with immature TLS (Figure 6G). Taken together, we found the TLS presence and heterogeneity in localization and maturation stages markedly differ between ccRCC patients with distinct predictive responses for immunotherapies.

Next, we further conducted additional analyses to investigate whether the combination of TLS localization and maturation heterogeneity can provide further insights into prediction for immune responses in ccRCC patients receiving ICIs treatments. The results obtained from this classification revealed noteworthy patterns in the response to ICIs. Notably, the intra‐/mature group displayed the highest objective response rates (CR/PR) after ICIs treatment, reaching up to 54.6% of patients. Subsequent analysis of the peri‐/mature, intra‐/immature, and peri‐/immature groups revealed objective remission rates of immune response at 44.5, 33.3, and 18.6%, respectively (Figure 6H). Moreover, Kaplan–Meier survival analysis indicated that patients within the intra‐/mature group exhibited significantly improved PFS (*p* = 0.025) and OS (*p* = 0.024) compared with other ccRCC patients with TLS who received ICIs postoperative adjuvant therapy (Figure 6I).

In the context of clinical benefit analysis following ICIs treatment, ccRCC patients were classified into three distinct groups: clinical benefit (CB), intermediate clinical benefit (ICB), and no clinical benefit (NCB). The outcomes highlighted distinct response rates among these groups (Figure 6J). Notably, the intra‐/mature group demonstrated CB and ICB rates of 72.7% and 18.2%, respectively, whereas the intra‐/immature group displayed CB and ICB rates of 61.2% and 33.3%, respectively. Contrastingly, the peri‐/mature group evidenced CB and ICB in 44.4 and 22.2% of patients, while the peri‐/immature group depicted a CB rate of only 29.6%, with a substantial proportion achieving ICB (44.4%).

Overall, the cumulative findings from this comprehensive analysis suggest that a combined assessment of TLS spatial distribution and maturity heterogeneity could serve as a more efficient indicator of ccRCC patients’ response to ICIs treatment. This integrated approach could significantly contribute to the accurate identification of the ccRCC patient population most suited for immunotherapeutic interventions.

### 2.7 IgA and IgG staining demonstrate significantly higher objective response rates and superior prognosis for ccRCC patients subjected to immunotherapy

**FIGURE 7:**
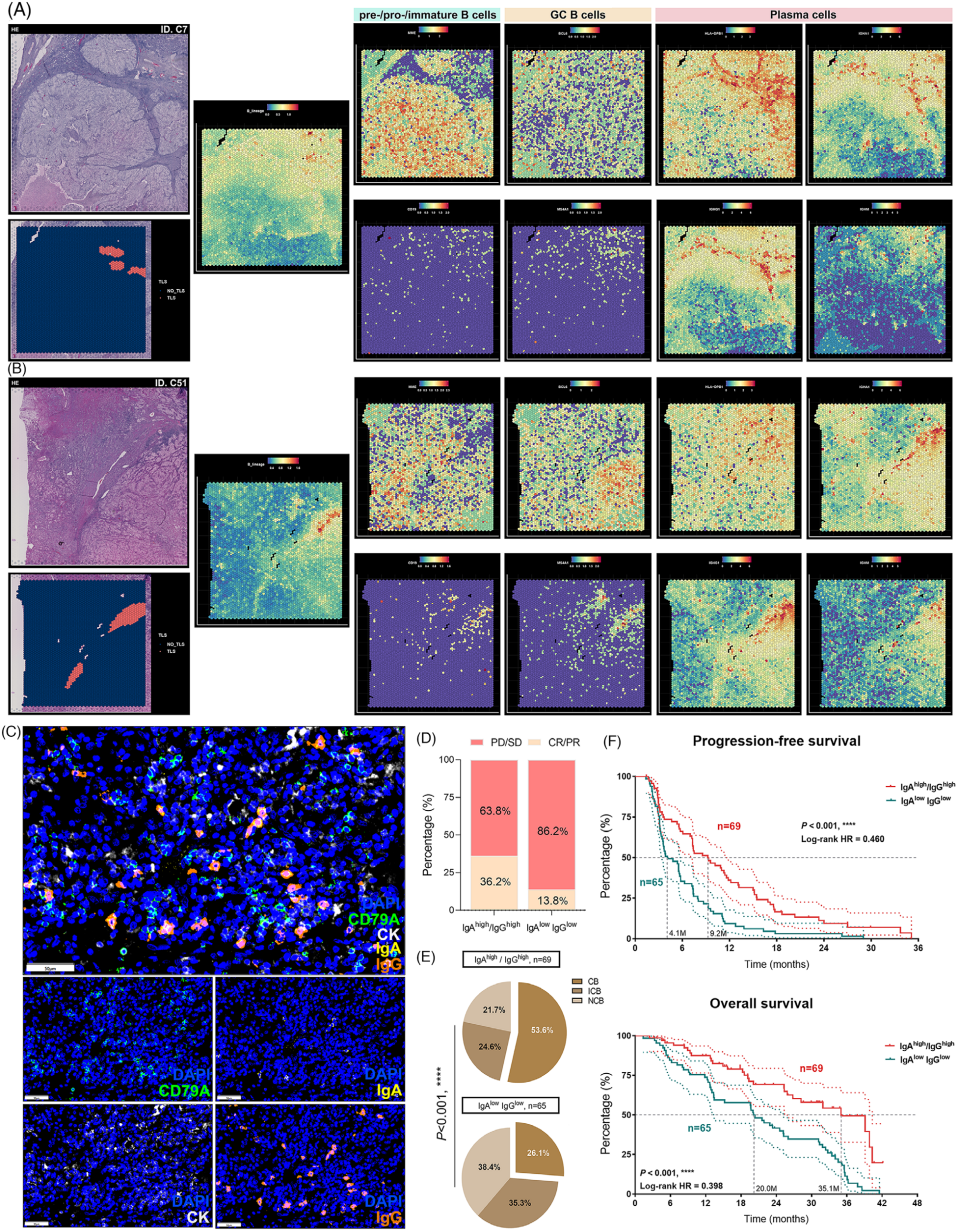
IgA and IgG staining correlated with higher immunotherapy response and superior prognosis for ccRCC patients. (A and B) The location and abundance of B cell and immunoglobulin‐associated genes within tumor‐associated TLS regions in two representative ccRCC tissue samples analyzed by spatial transcriptomic data. (C) Representative mIF staining (combining DAPI, CD79A, CK, IgA, and IgG) images of ccRCC tissue samples treated with ICIs within the FU‐ICIs cohort. Scale bar: 50 μm. (D) The percentage of PD/SD and CR/PR metastatic ccRCC patients in IgA^high^/IgG^high^ and IgA^low^/IgG^low^ groups. (E) The clinical benefit analysis in ccRCC patient groups following ICIs treatment. (F) Kaplan–Meier curve comparing OS and PFS between IgA^high^/IgG^high^ and IgA^low^/IgG^low^ ccRCC patients.

B cells residing within well‐formed, mature TLS serve multiple functions, including the release of antibodies against tumors and providing neoantigens to activate T cells within the TME, thereby augmenting the immune system's capacity to target tumor cells and enhancing the likelihood of a favorable response to immunotherapy. In light of spatial transcriptomic data obtained from ccRCC, we observed high expression of B cell and immunoglobulin‐associated genes within tumor‐associated TLS regions, as illustrated in two representative tissue samples (Figures 7A and B). Further scrutiny into B cell subtypes revealed that germinal B cells, primarily characterized by BCL6^+^/MS4A1(CD20)^+^, were prominently distributed in the TLS region, whereas the gene signatures of MME(CD10)^+^/CD19^+^ naive B cells showed lesser expression. Notably, the expression profiles of various immunoglobulins associated with plasma cells (with IgA and IgG being notably significant) differed markedly between TLS and tumor regions, with minimal presence in tumor tissues distanced from TLS (Figures 7A and B). This observation suggests that TLS might serve as the locale for the generation of IgA and IgG plasma cells. Moreover, an abundance of mature plasma cells within TLS has the capacity to produce Ig, dispersing along the fibroblast structure to various areas within the tumor tissue under the influence of chemokines.

The enrichment of IgA and IgG within the tumor environment is closely linked to the presence of TLS, with higher TLS maturity frequently associated with dense distribution of plasma cells and immunoglobulins within tumors. Nonetheless, the extent to which immunoglobulins independent of TLS variables can predict immunotherapy response necessitates further exploration. Consequently, we delved deeper into the types of immunoglobulins secreted by plasma cells and their correlation with the response of ccRCC patients to ICI therapy. Subsequently, utilizing mIF, we assessed the protein expression of IgA and IgG in 134 ccRCC tissue samples treated with ICIs within the FU‐ICIs cohort subset (Figure 7C). Samples exhibiting elevated expression of IgA and IgG immunoglobulins were categorized as IgA^high^/IgG^high^, while those displaying low expression of both IgA and IgG were defined as IgA^low^ IgG^low^. Within the sample of 134 patients, our findings showcased significantly higher rates of immunotherapy benefits in the IgA^high^/IgG^high^ cluster compared with the IgA^low^ IgG^low^ cluster (CR/PR: 36.2 vs. 13.8%) (Figure 7D), alongside a markedly higher assessment of complete CB in the former (CB: 53.6 vs. 26.1%; *p* < 0.001) (Figure 7E). Moreover, the results from survival analysis indicated that patients within the IgA^high^/IgG^high^ clusters experienced substantially reduced risk of recurrence in PFS (*p* < 0.001, HR = 0.460) and superior long‐term survival benefits (*p* < 0.001, HR = 0.398) (Figure 7F). These observations signify that tumors exhibiting high IgA and IgG staining demonstrate significantly higher objective response rates and superior prognosis for ccRCC patients when subjected to immunotherapy.

## 3 DISCUSSION

TLS are a “hot spot” in cancer research, specifically due to their relation to the prognosis of cancer treatment. Indeed, TLS have been characterized in many cancer types and mainly correlate with good therapeutic outcomes^30^, ^31^; however, there are limited reports about TLS in renal cell carcinoma. In the existing studies, the presence of TLS is contradictory.^18^, ^25^ Therefore, a more comprehensive analysis of clinical data and assessment of TLS characteristics are needed. In this study, we presented three key results that can be implemented in future clinical examinations and prediction of prognosis.

First, histology assessments of TLS revealed its heterogeneity in ccRCC. Studies on other cancers described two types of localization and three maturation stages of TLS.^13^, ^32^ We, herein, identified both intratumoral and peritumoral TLS in ccRCC. More interestingly, the intratumoral TLS in ccRCC were predominantly SFL‐TLS, while peritumoral TLS were mostly E‐TLS. This observation is different from the findings of Masuda et al.,^25^ that no primary or SFL‐TLS is found in ccRCC. Considering the relatively low presence of PFL‐TLS (7.2%) and SFL‐TLS (10.3%) of all ccRCC samples identified in this study, a possible explanation is that when patient numbers included in the study are limited and sample sectioning is small, they are less likely to be identified. However, the mechanisms of TLS maturation differences in different locations are unclear. The better recruitment of FDCs and lymphocytes in intratumoral TLS may be attributed to the abundant vasculature within ccRCC.^33^ Additionally, the presence of TLS and B cell signatures such as IgG predict favorable responses to ICIs therapy in ccRCC patients.^18^, ^34^, ^35^ And on the other hand, ICIs treatment was reported to induce TLS formation after treatment in combination with neoadjuvant.^36^ Thus, it is worth investigating the prognostic value of TLS in ccRCC patients treated with antiangiogenic drugs and combination therapy.

Second, statistical models suggested that intratumoral TLS and peritumoral TLS with different mature stages and localization correlate with opposite prognostic trends in ccRCC. This result may help explain the paradox of TLS function in renal carcinoma. Our data showed that the presence of TLS does not predict ccRCC prognosis but a further examination of TLS localization is of great clinical significance. Intratumoral TLS indicate a better prognosis, which is in line with the findings of Meylan et al. in ccRCC and what is generally recognized in other cancer types.^26^, ^37^, ^38^ While peritumoral TLS, which are mainly at the immature stage, are associated with poor prognosis in ccRCC, and are consistent with the observations of Masuda *et al*., the divergent value in prognosis highlighted the heterogeneity of TLS in ccRCC and the importance of characterization of TLS features.^25^ Interestingly, patients with peritumoral TLS showed a prominently higher risk of reoccurrence and decreased clinical outcomes in breast and liver cancers.^26^, ^39^ Recently, Fridman et al. proposed that peritumoral TLS may not be directly associated with tumor but self‐antigens from inflamed tissues and should not be taken into account to analysis. On the other hand, researches for other cancer types, such as colorectal and lung cancers, stressed the importance of having mature TLS in positive prognosis, which may add more pathological relevance to their function.^13^, ^29^, ^40^, ^41^ Since the intratumoral TLS that predict the better prognosis are predominantly mature TLS, investigating if TLS maturation could be the confounding reason for the difference in prognostic associations in ccRCC rather than the localization constitutes a possible goal for future research. Evidences suggest that E‐TLS may be initiated from T cell factor 1 (TCF‐1) ‐expressing stem‐like T cells, which may interact with CD4^+^CXCL13^+^ T helper cells and consequently attract B cells.^42^, ^43^ However, E‐TLS lack sufficient antigen presentation and therefore specific antitumor response are obstructed. While mature TLS (PFL‐TLS and SFL‐TLS) take the antitumor immunity a significant step further as characterized by the FDC network. During the transition from PFL‐TLS to SFL‐TLS, a GC occur and enables the maturation of B cells to plasma cells, which produce hypermutant, high‐affinity antibody, mainly IgG and IgA. Indeed, IgG^+^ plasma cells and IgG coated tumor cells were observed in ccRCC samples, indicating the dominant role of B cells in TLS function and tumor‐rejection.^18^ The formation and maturation of TLS are likely to be associated with a series of chemokines such as CXCL12, CXCL13, CCL19, and CCL21. For example, tissue‐specific expression of CXCL13 induced B‐cell aggregation lacking the FDC network^44^; whereas expression of TNF and CXCL12 induced a TLS dominated by B‐cells supplemented by a small number of T‐cells as well as DCs.^25^, ^45^ Therefore, further investigation how the TLS mature will be of great clinical interest.

Third, multispectral fluorescence IHC (mIHC) staining of immune cells further dissected the differences in tumor environment between intratumoral and peritumoral TLS in ccRCC. We found higher stromal PD‐L1^+^ M2 macrophage and tumoral Treg infiltration in peritumoral TLS relative to intratumoral TLS, and both cells represent an immune suppressive environment.^46^, ^47^ Additionally, we found a higher tumoral PD‐1^+^CD8^+^ T cell infiltration in peritumoral TLS than in intratumoral TLS. Present with the PD‐L1^+^ M2 macrophage and Treg, these T cells are likely to be exhausted and are reported to correlated with immune‐cold TME status or adaptive immune resistance.^46^, ^48^, ^49^ Together this may explain the poor prognosis of peritumoral TLS of ccRCC and attempts to reinvigorate the PD‐1^+^CD8^+^ T cells and challenge the suppressive TME may increase patients’ sensitivity to the current immunotherapy. Other researchers found the pre‐existing tumor immunity in RCC predicts better response to ICIs therapy, which may occur in the APC‐dense niche in the TME.^50^ It will be interesting to take the APC‐dense niche and TCF‐1^+^ CD8 T cells into the future assessment, especially when examining the SFL‐TLS.

Forth, interpretation of the spatial transcriptomics data from a previous study by Meylan et al. revealed the aggregations of B cells, particularity plasma cells, the enrichment of a series of chemokines such as CXCL13, CCL19 in TLS and the enrichment of CAFs surrounding TLS. Additionally, NK cells and cytotoxic T cells were also increased around TLS region. Together, this suggests the formation and maturation of TLS as well as the antitumor reaction stimulated by them. As suggested by Fridman et al., TLS induce in situ antitumor immunity predominantly through high‐affinity antibodies with the help of macrophages or NK cells.^14^, ^32^ Moreover, the exclusive expression of CXCL9 and SPP1 in TAMs within ccRCC samples corroborates with the findings in head and neck squamous cell carcinoma. It was suggested that the CXCL9/SPP1 enrichment, rather than the M1/M2 markers, defines the polarity of TAMs in vivo.^51^ Therefore, it will be intriguing to explore the association of CXCL9 and SPP1 expression with TLS maturation and their prognostic value in ccRCC.

There are still some limitations in this study: (1) tumor‐specific TLS provide an essential microenvironment for immune cells aggregation and humoral immune response, so TLS are expected as a promising signature to effectively predict ICI tolerance in patients with solid tumors. However, this study has not explored the predictive value of TLS in ICI response for patients with advanced ccRCC. (2) Although a large number of patients with ccRCC were included in this study and the cohorts were independent from each other, the nature of retrospectives could not be avoided, which would reduce the clinical significance of our work. Therefore, future prospective cohorts are needed to verify the hypothesis proposed in this study. (3) This work failed to assess the association between the ratio of TLS to the tumor area and ICIs treatment response. The H&E sections encompassed ccRCC tumor tissue along with adjacent normal tissue, yet did not entirely encapsulate the entire spectrum of the tumor tissue within the field of view. Consider incorporating an analysis of the ratio of TLS to tumor area to explore its potential impact on ICIs treatment response in ccRCC patients could be a promising future direction. (4) Currently, therapies involving induced TLS are mainly used in preclinical studies of cancers, and are increasingly being viewed as a promising new strategy for cancer treatment. For example, antiangiogenesis therapy combined with PD‐L1 blocking can regulate angiogenic vascularity, induce TLS, and enhance cytotoxic T cell activity, resulting in additional survival benefits.^52^ This study failed to further explore the mechanism of TLS localization and maturation heterogeneity in effecting abnormal TME phenotypes of ccRCC, and we have not yet carried out studies on the TME mimicking ccRCC in vivo. The exploration in immunosound in situ xenografted tumor model or PDO/PDX model may be the next research direction in the future.^53^

In conclusion, this study revealed the predictive value of TLS heterogeneity in maturation status and localization on the progression and immunological responses of ccRCC for the first time. The morphology of most TLS reside in the peritumoral area tends to exhibit lower maturity. The intratumoral, mature TLS could be used to evaluate favorable prognostic patterns and the unique TME characteristics of each patient. TLS might serve as the locale for the generation of IgA and IgG plasma cells, demonstrating significantly higher objective response rates and superior prognosis for ccRCC patients subjected to immunotherapy. These findings would allow the identification of immunophenotypes and the improvement of immunotherapeutic effectiveness for ccRCC.

## 4 MATERIALS AND METHODS

### 4.1 Clinical sample collection of the integrated FUSCC cohort and the FU‐ICIs cohort

We established an integrated cohort encompassing 429 patients diagnosed with ccRCC who underwent either radical or partial nephrectomy at the Department of Urology, FUSCC (Shanghai, China). The recruitment period spanned from August 2009 to October 2022 for those who received surgical intervention or from August 2012 to April 2023 for individuals seeking pathological consultation at the FUSCC pathology department. Only patients with complete pathology reports and electronic medical records were considered for inclusion (the Integrated FUSCC cohort). Exclusion criteria comprised cases with incomplete medical records, inadequate follow‐up information, confirmed nonclear cell carcinoma components, or preoperative exposure to adjuvant therapies. Additionally, paired tumor and adjacent nontumor tissues were mandatory for inclusion, with the absence of adjacent normal tissue leading to exclusion from the study.

The FU‐ICIs cohort, constituting 230 ccRCC patients, received combined therapy involving TKIs and ICIs from January 2016 to December 2021. Therapeutic response and disease progression were assessed following the RECIST 1.1 criteria. Postoperative tissue specimens were histopathologically identified as ccRCC and collected from the FUSCC tissue bank after fixation in 4% paraformaldehyde.

Key clinicopathological indicators analyzed included the patients' age at initial diagnosis, gender, pathological TNM stage, AJCC stage, and ISUP grade. Table S1 details the clinicopathological characteristics at baseline for the 429 patients in the integrated FUSCC cohort.

### 4.2 H&E and IHC assays

The analysis involved the histopathological assessment of unspecific lymphocytic infiltration on H&E slides using established protocols,^54^ conducted independently by two experienced pathologists. Selected ccRCC tissue or adjacent normal kidney tissues were chosen for H&E staining, which allowed assessment of TLS localization. Specimens exhibited potential microscopic TLS after H&E staining culminating in successive ccRCC specimens slices confirmed to bear definite TLS presence via IHC identification. TLS were then identified as lymphocyte aggregates resembling lymphoid tissues with B cells (CD20, ab64088; Abcam), T cells (CD3, ab16669; Abcam), FDCs (CD21, ab7290; Abcam), and GC cells (CD23, ab16702; Abcam) within the intratumoral or adjacent peritumoral regions using subsequent IHC staining (n = 169). Identification of TLS positivity from ccRCC slides involved the recognition of at least one TLS, categorized by location heterogeneity. Intra‐TLS clusters were defined within the tumoral invasive margin, while peritumoral TLS (peri‐TLS clusters) were identified within normal tissue over 10 mm outside the invasive margin.^13^ This determination was made independently by a urological surgeon and two pathologists. According to the spatial identification of TLS, the TLS‐positive samples were categorized as follows: intra‐TLS samples (intra‐TLS cluster) had at least one intratumoral TLS, peri‐TLS TLS samples (peri‐TLS cluster) had at least one peritumoral TLS but no intratumoral TLS, and “both” samples had both peritumoral and intratumoral TLS (a subset of the intra‐TLS cluster).

For TLS maturation analysis, slides were co‐stained for CD3, CD20, CD21, and CD23.^28^, ^41^, ^55^ High‐power field (HPF) images of all dense lymphocytic aggregates, regardless of CD3/CD20/CD21/CD23 signals were captured. Each HPF image was examined to ascertain the TLS maturation stage: E‐TLS, dense lymphocytic aggregates with mixed B and T cells lacking CD21 and CD23 signals (no FDC, no GC); PFL‐TLS, dense lymphocytic aggregates with CD21 but no CD23 signals (FDC, but no GC); SFL‐TLS, characterized by the presence of CD21^+^CD23^+^ FDCs, indicating the existence of a GC (both FDC and GC reaction).^28^, ^41^, ^55^, ^56^ TLS‐positive samples were then classified based on the stages of TLS maturation: SFL‐TLS samples had at least one SFL‐TLS, PFL‐TLS samples had at least one PFL‐TLS but no SFL‐TLS, and E‐TLS samples had neither PFL‐TLS nor SFL‐TLS.^56^

### 4.3 Exploring the role of TLS in TME of ccRCC with spatial transcriptomics analysis

Maxime Meylan et al. explored the associations between intratumoral TLS and patients’ clinical outcome by using spatial transcriptomics in a previous study.^18^ To further explored the role of TLS in TME of ccRCC, we obtained the spatial transcriptome datasets published by them from Gene Expression Omnibus (GEO; accession ID: GSE175540). The gene‐spot matrices obtained through the processing of spatial transcriptomics and visium samples were analyzed using “Seurat” package in the R software.^57^ Spots with a minimum gene count of 200 were selected while genes with read counts below 10 or expression in fewer than 10 spots were excluded. Dimensionality reduction and clustering using independent component analysis (PCA), considering the first 30 principal components and spatial feature expression plots were created using the SpatialPlot function within the Seurat package. As Meylan et al. have provided the TLS annotations in the datasets, we added the TLS information by modified the metadata of the Seurat object. In addition, MCPcounter^58^ was utilized to make deconvolution and quantify the various cell types in the spatial transcriptome datasets. Based on the spatial transcriptome data, we explored the potential location of TLS and also explored the immune and stromal cell spatial population and expression level of related chemokines which may play a key role in the formation or maturation of TLS in ccRCC microenvironment.

### 4.4 mIHC and mIF staining assays

The mIHC process utilized the Akoya OPAL Polaris 7‐Color Automation IHC kit (NEL871001KT) with two distinct panels (Panel 1: CD8, CK, CD68, CD163, PD‐1, PD‐L1, DAPI; Panel 2: CD3, CK, CD56, CD20, CD4, FoxP3, and DAPI) as depicted in Figure 4A. First, a total of 63 tissue samples were initially deparaffinized in a BOND RX system (Leica Biosystems) and subjected to staining with specific primary antibodies.^59^, ^60^, ^61^ The slides, following the primary antibody incubation, were then treated with secondary antibodies labeled by horseradish peroxidase (HRP) and corresponding reactive Opal fluorophores based on the tyramide signal amplification principle.^60^, ^61^ Slides bound with primary and secondary antibodies but excluding fluorophores served as negative controls to gauge autofluorescence. These multiplex stained slides were scanned through the Vectra Polaris Quantitative Pathology Imaging System (Akoya Biosciences) at 20 nm wavelength intervals from 440 to 780 nm, producing a single image using inForm v.2.4.8 (Akoya Biosciences) to quantify the different biomarkers in each cell.^60^, ^61^ The quantity of various cell populations was expressed as both the number of stained cells per square millimeter and as the percentage of positively stained cells among all nucleated cells, following the manufacturer's instructions.^59^, ^60^, ^61^

For the evaluation of DCs in relation to TLS maturation, 75 ccRCC slides were co‐stained for CD20, CD21, CD23, CD45, DC‐LAMP, pan‐CK, and DAPI. All dense lymphocytic aggregates and areas exhibiting DC‐LAMP signal were imaged and analyzed using seven‐color mIF.^28^ This process detected DC‐LAMP^+^ (DC‐LAMP/CD208; Dendritics; DDX0191) mature antigen‐presenting DCs and other classic markers present in TLS at different maturation stages with varying frequencies.^62^, ^63^

The mIF analysis involved deparaffinizing 134 paraffin sections and then conducting antigen epitope retrieval while blocking nonspecific binding sites with antibody diluent. The slides underwent incubation with primary antibodies, secondary polymer HRP (mouse and rabbit), and signal amplification. Each experiment included an isotype control slide that omitted the primary antibody. Specific proteins were detected using the following antibodies: anti‐CD79A (#ab238096, 1:100; Abcam), anti‐IgA (#ab193189, 1:100; Abcam), anti‐IgG (#ab109489, 1:100; Abcam), and pan‐CK (#ab7753, 1:100; Abcam).

### 4.5 Statistical analysis

The statistical and graphical analyses were performed using the SPSS software (version 23.0), GraphPad Prism software (version 8.0), or R software (version 3.3.2). One‐way analysis of variance was performed to conduct comparisons among multiple groups (≥2). The Student's *t*‐test was used to assess the statistical differences between the two groups.

## AUTHOR CONTRIBUTION

*Conceptualization*: W. Xu, J. Lu, X. Tian, W. Liu, and H. Zhang. *Data curation and formal analysis*: W. Xu, J. Lu, X. Tian, S. Ye, and S. Wei. *Funding acquisition*: W. Liu, K. Chang, H. Zhang, and D. Ye. *Investigation and methodology*: W. Xu, J. Lu, X. Tian, W. Liu, S. Ye, J. Wang, and S. Wei. *Resources and software*: J. Wang, S. Wei, Y. Qu, K. Chang, H. Zhang, and D. Ye. *Supervision*: K. Chang, H. Zhang, and D. Ye. *Validation and visualization*: W. Xu, J. Lu, W. Liu, and X. Tian. *Original draft*: W. Xu, X. Tian, and J. Lu. *Editing*: W. Liu, K. Chang, H. Zhang, and D. Ye. All authors have read and approved the final manuscript.

## ETHICS STATEMENT AND CONSENT TO PARTICIPATE

The study design and experimental procedures for this study followed the Helsinki Declaration II. Written Informed Consent were obtained from all participants. This study was approved by the ethics committee of Fudan University Shanghai Cancer Center (No: 050432‐4‐2108*, FUSCC, Shanghai, China).

## CONFLICT OF INTEREST STATEMENT

The authors report no potential conflicts of interest.

## Supporting information

Supplementary information

## Data Availability

The datasets used and/or analyzed in the study are available in the supplements or from the corresponding author upon reasonable request.
