# Supplementary material for: Unveiling the impact of tertiary lymphoid structures on immunotherapeutic responses of clear cell renal cell carcinoma
Source: MedComm (2020). 2024 Jan 12;5(1):e461. doi: 10.1002/mco2.461 (PMC10784869; doi:10.1002/mco2.461)
Supplement: Supplementary file 1 — Supplementary information [file MCO2-5-e461-s001.docx]

**Unveiling the Impact of Tertiary Lymphoid Structures on Immunotherapeutic Responses of Clear Cell Renal Cell Carcinoma**

**Running title:** TLS predicts responses to immunotherapy of ccRCC

Wenhao Xu^1,2^^#^, Jiahe Lu^1,2,3#^, Xi Tian^1,2#^, Shiqi Ye^1,2#^, Shiyin Wei^4#^, Jun Wang^5^, Aihetaimujiang Anwaier^1,2^, Yuanyuan Qu^1,2^, Wangrui Liu^6^*, Kun Chang^1,2^*, Hailiang Zhang^1,2^*, Dingwei Ye^1,2^*

# Contribute equally to this work

**Author affiliations**

^1^ Department of Urology, Fudan University Shanghai Cancer Center; Department of Oncology, Shanghai Medical College, Fudan University, Shanghai, 200032, P.R. China

^2^ Shanghai Genitourinary Cancer Institute, Shanghai 200032, P.R. China.

^3^ School of Cellular and Molecular Medicine, University of Bristol, Bristol BS8 1TD, UK.

^4^ Affiliated Hospital of Youjiang Medical University for Nationalities, Baise, 533000, P.R. China

^5^ State Key Laboratory of Oncology in South China, Collaborative, Innovation Center for Cancer Medicine, Department of Urology, Sun Yat-sen University Cancer Center, Guangzhou 510060, P.R. China.

^6^ Renji Hospital, Shanghai Jiao Tong University School of Medicine, Shanghai 200127, P.R. China.

***Corresponding authors**

Prof. **Dingwei Ye** MD. (Email: [dingwei_ye@fudan.edu.cn)](mailto:dingwei_ye@fudan.edu.cn))

Prof. **Hailiang Zhang** MD. (Email: [zhanghl918@163.com](mailto:zhanghl918@163.com))

Prof. **Kun Chang** MD. (Email: [changkungene@126.com)](mailto:changkungene@126.com))

Department of Urology, Fudan University Shanghai Cancer Center

Department of Oncology, Shanghai Medical College, Fudan University

Shanghai, 200032, P.R. China

Dr. **Wangrui Liu** MD. (Email: [cowdl@163.com)](mailto:quyy1987@163.com))

Renji Hospital, Shanghai Jiao Tong University School of Medicine

Shanghai 200127, P.R. China.

**Supplementary Figure legends**

**Figure S1. Larger overviews of HE-stained slices.** Each section for HE was required to contain both ccRCC and para-cancer normal tissue. In the determination of TLS location heterogeneity, at least one TLS in HE slices was confirmed to be TLS positive. TLS was defined as intratumoral TLS when it was located within the invasive margin of the tumor, and peritumoral TLS when it was located in normal tissue more than 10mm outside the invasive margin. This identification process was performed independently by a urological surgeon and two pathologists.

**Figure S2. Heterogeneity of TLS maturation and cellular components assessed by multispectral fluorescent immunohistochemistry (mIHC)** **at low magnification.** Cellular composition of TLS in heterogeneous ccRCC samples was detected using mIHC in 63 cases of ccRCC tissues with two sets of 7-marker multispectral fluorescent immunohistochemistry applied for further analysis at low magnification.


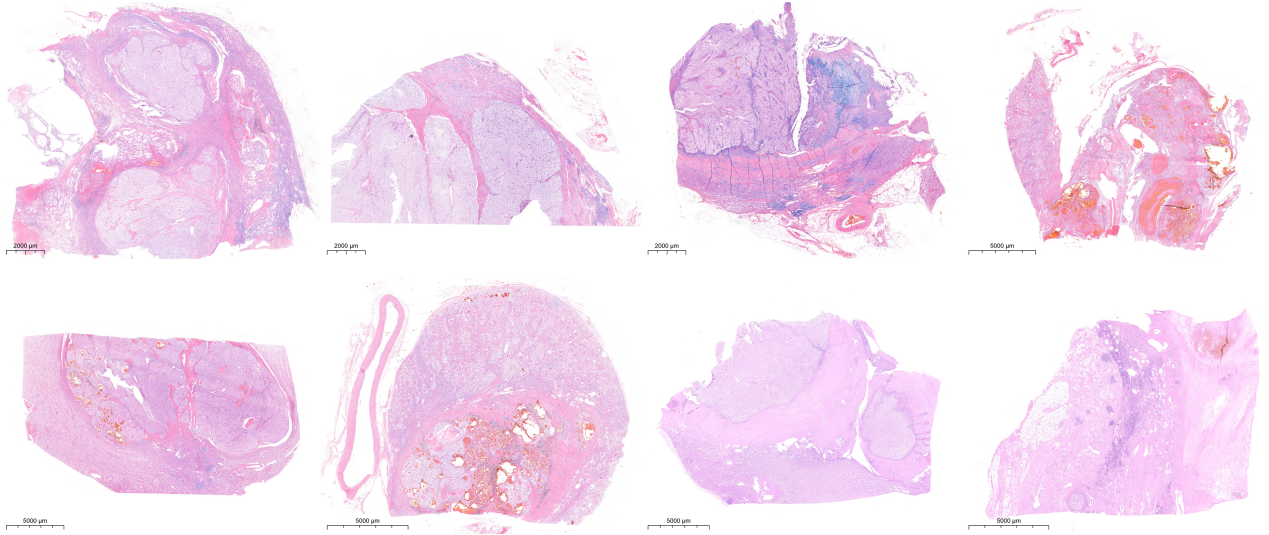


**Figure S1. Larger overviews of HE-stained slices.**


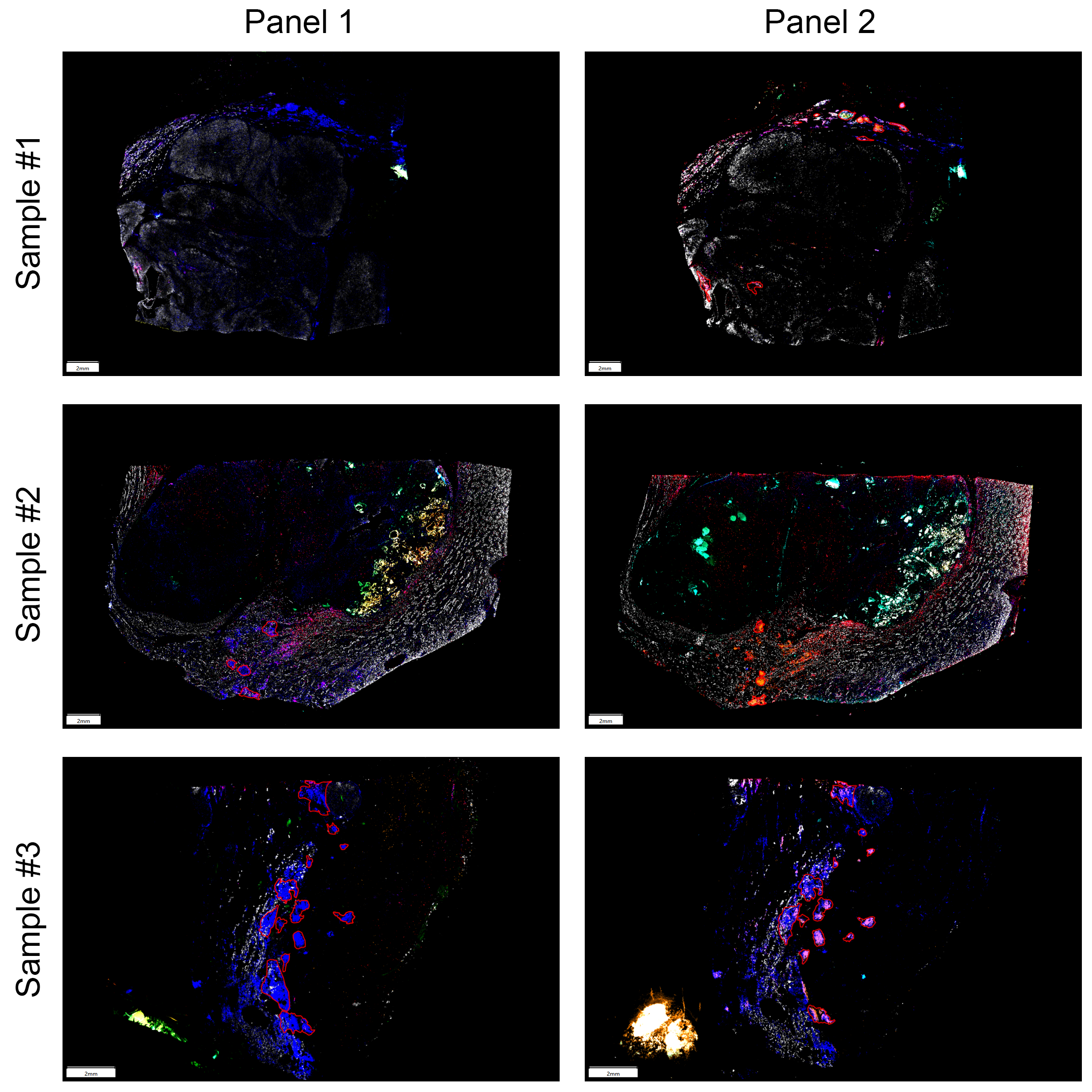


**Figure S2. Heterogeneity of TLS maturation and cellular components assessed by multispectral fluorescent immunohistochemistry (mIHC)** **at low magnification.**

**Table S1. Clinicopathological characteristics of 429 patients with ccRCC from the integrated FUSCC cohorts.**

| Clinicopathological Characteristics | Integrated FUSCC cohorts (n=429) |
| --- | --- |
|  | No. (%) |
| Age |  |
| ≥60 years | 241 (56.2) |
| <60 years | 188 (43.8) |
| Gender |  |
| Male | 268 (62.5) |
| Female | 161 (37.5) |
| pT stage |  |
| T1-T2 | 326 (76.0) |
| T3-T4 | 103 (24.0) |
| pN stage |  |
| N0 | 343 (80.0) |
| N1 | 86 (20.0) |
| pM stage |  |
| M0 | 343 (80.0) |
| M1 | 67 (15.6) |
| AJCC stage |  |
| I- II | 307 (71.6) |
| III-IV | 122 (28.4) |
| ISUP grade |  |
| G1-G2 | 241 (56.2) |
| G3-G4 | 188 (43.8) |
| TLS |  |
| Presence | 145 (33.8) |
| Absence | 284 (66.2) |
| Localization |  |
| Peri-TLS | 103 (71.0) |
| Intra-TLS | 42 (29.0) |
| Maturation |  |
| E-TLS (Immature TLS) | 69 (47.6) |
| PFL-TLS(Immature TLS) | 31 (22.8) |
| SFL-TLS (Mature TLS) | 44 (30.3) |

(FUSCC, Fudan University Shanghai Cancer Center; AJCC, the American Joint Committee on Cancer; TNM stage, Tumor size, Lymph Nodes affected, Metastases; ISUP: International Society of Urological Pathology; **^※^**P value less than 0.05 was marked in bold)
